# Reproductive Disorders in Donkeys: Current Evidence and Update

**DOI:** 10.3390/ani14172494

**Published:** 2024-08-28

**Authors:** Zixuan Wang, Shenming Zeng, Yantao Wang, Tao Wang, Honglei Qu, Yiping Zhu, Jing Li

**Affiliations:** 1 Equine Clinical Diagnostic Center, College of Veterinary Medicine, China Agricultural University, Beijing 100193, China; zixuanw@cau.edu.cn; 2 National Key Laboratory of Veterinary Public Health and Safety, College of Veterinary Medicine, China Agricultural University, Beijing 100193, China; 3 College of Animal Science and Technology, China Agricultural University, Beijing 100193, China; zengsm@cau.edu.cn; 4 National Engineering Research Center for Gelatin-Based Traditional Chinese Medicine, Dong-E-E-Jiao Co., Ltd., Liaocheng 252200, China; wangyt@dongeejiao.com (Y.W.);; 5 Shandong Dong-E Black Donkey Animal Husbandry Technology Co., Ltd., Dong-E-E-Jiao Co., Ltd., Liaocheng 252200, China; 2017305010305@cau.edu.cn

**Keywords:** donkey, reproductive disease, uterine disease, abortion

## Abstract

**Simple Summary:**

Donkeys play crucial roles in agriculture and human livelihoods globally. Reproductive disorders in donkeys pose significant challenges to their health and well-being. While donkeys share many similarities with horses, specific aspects of donkey disorders remain poorly understood. Knowledge about reproductive disorders of donkeys is important for accurate diagnosis and effective treatment for these animals. This review aims to provide a brief update on reproductive disorders of donkeys including common infectious and non-infectious causes for infertility and pregnancy loss. Understanding reproductive health in donkeys plays a crucial role in enhancing the welfare status of donkeys and decreasing associated economic loss.

**Abstract:**

Reproductive disorders in donkeys present a significant challenge to their health and welfare, impacting their roles in agriculture, conservation, and companionship. With the development of large-scale donkey farming in recent years, reproductive disorders have become a limiting factor for the expansion of the donkey population. In general, donkeys suffer from a similar array of diseases like horses, but little is known about the specificities of donkey reproductive disorders. This review synthesizes current knowledge on the pathogenesis, distribution, presentation, diagnosis, treatment, and prognosis of a diverse array of reproductive disorders affecting donkeys. There are similar infectious and non-infectious causes for infertility and pregnancy loss in jennies compared with mares, but a difference in disease susceptibility does exist, which may be attributed to genetic influence, pathogen specificity, the environment, and reproductive management practices. Diagnostic and treatment plans need to be tailored towards the particularities of donkey reproductive disorders to increase donkey populations and to enhance the standard of care for this species. Enhancing reproductive health in donkeys not only ensures their sustainable use but also promotes their welfare and longevity in diverse human–animal interactions.

## 1. Introduction

There has been a 26% increase in the number of donkeys worldwide from 1997 to 2022, and the current estimate of the world donkey population is 51.7 million [1]. Asia and Africa are the two continents with the largest number of donkeys, and the top five countries are Ethiopia, Sudan, Chad, China, and Burkina Faso, each with over 1 million donkeys in stock [2]. In the past few decades, large-scale donkey farming systems have been developed with the increased demand for donkey products. Some breeding farms in China can house from hundreds to thousands of donkeys [3].

The reproductive performance of the donkeys has become a crucial aspect of the productivity and sustainability of the donkey industry. In recent years, outbreaks of reproductive disorders in large-scale systems have posed significant challenges to the reproductive efficiency of the breeding population, which has exacerbated the shortage of supply on local markets. In addition, many donkey breeds in Europe are becoming endangered due to industrialization, and conservation efforts have been made through the introduction of breeding programs [4]. In areas where donkeys are still used as work animals, the fertility of the animals is also of great significance as they contribute to the daily production of local households and the stability of the communities [5]. Therefore, there is an urgent need globally for a better understanding of the specificities of donkey reproductive health.

**Figure 1:**
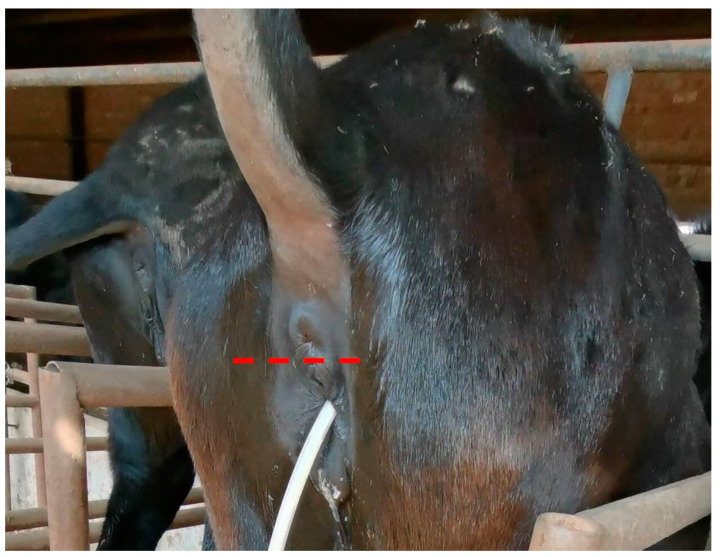
Perineal conformation of the jenny, note the entire vulva is below the pelvic brim (red dash line). An intrauterine tube was used to flush the uterus.

The reproductive anatomy and physiology of the donkey are similar to that of the horse, but it is necessary to realize certain unique characters of donkeys in order to accurately distinguish pathology from the natural variation of the species. One of the most distinct reproductive properties of the donkey is that the jennies (female donkeys) exhibit non-seasonal polyestrous cycles with no anestrous period like the mare [6]. The length of the estrous cycle in jennies appears to be slightly longer, more variable, and breed-dependent, typically ranging from 21 to 28 days [7,8]. The gestation length of the jennies has been reported to be longer than mares, averaging 353 to 371 days depending on the breed [7,9,10]. Unlike mares, the entire vulva of the jennies lies entirely below the pelvic brim (Figure 1) [11]. The ventral commissure of the vulva is usually located more cranial than the dorsal commissure, resulting in the ventral tilting of the vulvar vestibule [6]. The cervix of the jenny is narrowed, tortuous, and more protruded compared with the mare, posing difficulty for intrauterine manipulation and adding risks for cervical laceration during parturition [6]. In addition, uterine edema appears less prominent in jennies compared with mares, and therefore teasing as well as follicle size should be considered together for prediction of ovulation [6]. It is important to acknowledge these differences to prevent the misinterpretation or misdiagnosis of reproductive diseases in donkeys.

To date there has been little information regarding the reproductive disorders of the donkey. As an Equidae species, donkeys suffer from a similar series of infectious and non-infectious reproductive disorders, but the treatment options are usually extrapolated from horses due to insufficient relevant data. As issues have arisen worldwide with regard to appropriate care and welfare for this species, reproductive health is one crucial aspect to consider, especially in the scenario of conservation and the long-term sustainability of the species. This review aims to provide a comprehensive summary of the current evidence and knowledge about the classification, distribution, and therapy of the reproductive disorders of the donkey.

## 2. Materials and Methods

The literature search was conducted up to June 2024 with the following databases: PubMed, Google Scholar, and CNKI (China National Knowledge Infrastructure). For the literature search, the keywords in each type of disorder (for example, “endometritis”, “post-breeding endometritis”, and “persistent mating-induced endometritis”) were used in combination with “donkey”, “asinine”, “jenny”, and “jack” in the query. Boolean operators AND, OR, and NOT were utilized in the search. Our research primarily focused on the peer-reviewed literature in English and Chinese. The titles were screened and irrelevant publications were excluded from the literature pool. The studies related to donkeys, jennies, or jacks were considered as core references. All study types, including case reports, were included in the search as sources may be scarce on certain topics. The abstracts and conclusions of these studies were read thoroughly to ensure the soundness of the research design. The main text of the studies was also checked if detailed information was required for the integrity of this review.

The photographic resource of donkey reproductive disorders in the peer-reviewed literature is limited. To enhance the visual illustration of donkey reproductive disorders, a few classic pictures were incorporated in this review, which were taken by the authors during clinical and research work at donkey farms in China.

## 3. Reproductive Disorders of the Jenny

### 3.1. Uterine Disorders

#### 3.1.1. Persistent Mating-Induced Endometritis

Jennies experience a similar uterine inflammatory process post-breeding to mares [12]. The transient inflammation is a normal physiologic response to the introduction of foreign material including spermatozoa, microorganisms, and debris during mating [13]. Post-breeding endometritis is generally considered pathologic when the inflammation persists over 24–48 h post-mating [14]. This condition is generally known as persistent mating-induced endometritis (PMIE), which is characterized by an excessive influx of polymorphonuclear leukocytes (PMNs) into the uterine lumen and endometrium with accumulation of inflammatory material in the uterus. The molecular mechanism of post-breeding endometritis in mares has been investigated quite extensively. The local innate immune response is activated shortly after antigen introduction and recognition, and pro-inflammatory cytokines are secreted [13]. As part of innate immunity, neutrophils can exert antimicrobial functions by phagocytosis or by forming neutrophil extracellular traps (NETs) which contains extracellular DNA and proteins including histones, myeloperoxidase, cathepsin G, and elastase [15]. Although NET formation helps with eliminating infectious agents and possibly plays a role in the sperm selection process, its persistence may cause tissue damage and fibrosis development [13,15].

Like in the mares, uterine cytology (samples acquired from double-guarded swabs, cytobrushes, or low-volume uterine lavage) and uterine biopsy/histopathology are equivalently useful in diagnosing endometritis in the jennies [6]. Interestingly, eosinophils make up a characteristic population in this acute inflammatory response in the jennies, whereas neutrophils are the main PMN cell type found in mares [16]. In donkeys, artificial insemination with frozen-thaw semen elicits a more prominent endometrial inflammation than in mares [17]. No difference has been seen in the effect of vitrified and frozen-thawed semen on uterine inflammation, and therefore the presence of permeable cryoprotectants (i.e., glycerol) seems not to be the main cause of the decreased fertility of frozen semen [8]. The role of seminal plasma (SP) in endometritis of jennies has been investigated, and one study showed that the presence of SP did not reduce the PMN number but reduced the COX-2 expression after insemination with frozen-thawed semen [18]. An in vitro study indicated that SP instead of spermatozoa is able to induce NET functioning from jenny PMNs [12]. An in vitro model suggested that SP can suppress sperm–PMN binding, and specific SP fractions may be involved in this process in the donkey uterus [19,20].

PMIE can cause infertility as the presence of excessive inflammatory fluid within the uterus interferes with pregnancy establishment [21]. It has been a common practice to administer anti-inflammatory drugs to mares predisposed to PMIE around insemination [14]. The efficacy of steroids and non-steroidal anti-inflammatory drugs including dexamethasone, ketoprofen, vedaprofen, and firocoxib in reducing post-breeding inflammation has been investigated in mares [22,23,24]. In contrast, there are only a few studies with small animal groups on the medical treatment options for jennies with post-breeding endometritis (Table 1). A 5-day protocol of ketoprofen has been shown to inhibit COX-2 but not decrease PMN counts in the uterine cytology and biopsy samples [25]. A single dose of dexamethasone at insemination did not decrease PMN but decreased eosinophils 24 h post-AI [26]. With the development of equine regenerative medicine in recent years, the immunomodulatory effects of novel agents like platelet-rich plasma (PRP) have shown great potential in regulating the reproductive health of mares and jennies [27]. Intrauterine infusion of autologous PRP has been shown to reduce uterine wall thickness and promote restoration of normal endometrial morphology in jennies with acute endometritis [28]. Further research is warranted to clarify the therapeutic effects of different treatments in improving pregnancy rates in jennies with endometritis.

Poor perineal conformation has been proved to be a major contributing factor of endometritis in mares, which may be congenital or acquired in nature [15]. Acquired poor perineal conformation from post-partum rupture of the perineal structures leading to infertility has been reported in two Catalan jennies [30]. On the other hand, poor congenital perineal conformation is much less frequent in the case of jennies compared with mares [6]. The relative location of the vulva to pelvic brim, the downward slope of the pelvis, and the ventral tilting of the vulva in the jennies contribute to a more desirable perineal conformation that facilitates the evacuation of abnormally accumulated intrauterine fluid [31]. Therefore, the occurrence of endometritis in the jennies may be more commonly related to reaction to the semen and inadequate breeding management practices. To prevent endometritis from happening, it is vitally important to adhere to strict hygiene standards before performing any manipulation of the internal genital tract of the jennies [32].

#### 3.1.2. Infectious Endometritis

**Figure 2:**
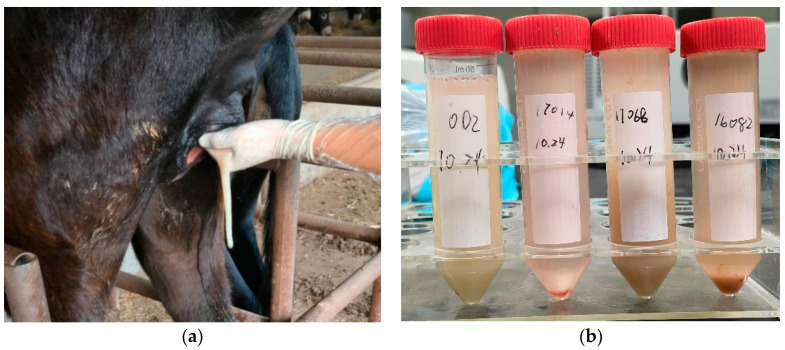
Purulent vaginal discharge in a jenny with bacterial endometritis. (**a**) Purulent discharge upon cervical manipulation; (**b**) appearance of low-volume uterine lavage samples (centrifuged) of jennies with bacterial endometritis.

Infectious endometritis of the equids is caused by bacterial or fungal agents. Uterine fluid accumulation is common, and diagnosis can be made based on positive uterine culture, increased white blood cells, and the presence of bacteria on cytology exam [33]. Purulent vaginal discharge can sometimes be seen (Figure 2a), and uterine lavage may recover cloudy fluid with tissue debris (Figure 2b).

The epidemiology of bacterial endometritis in jennies has been scarcely described. Based on the current evidence, its prevalence appears to vary upon farms and locations. One study found that 17 out of 84 (20.2%) jennies in the breeding population on a donkey farm in eastern China were diagnosed with bacterial endometritis with purulent vaginal discharge [34]. *Streptococcus equi* subsp. *zooepidemicus* (SEZ) was detected in the vaginal discharge by blood agar culture and PCR in all symptomatic cases [34]. Another study conducted in northern China showed that 65 to 100% of infertility in jennies was caused by bacterial endometritis confirmed by bacterial culture and PCR of the uterine swabs [35]. The bacterial agents causing endometritis in jennies have been isolated in several studies (Table 2). SEZ and *Escherichia coli* (*E. coli*) are the most commonly isolated bacteria from the uterine samples of jennies with endometritis [28,34,35,36], which corresponds well with the results from mares [37]. Mixed infection appeared to be more common than single infection [36]. Specific strains of SEZ have been reported to express super-antigens that permit them to persist in the uterine glands of mares [38]; however, the infection status of such strains has not been determined in jennies. Recently, a novel SEZ strain has been reported to cause severe bronchopneumonia in donkeys, suggesting the potential pathogenicity of SEZ in this species [39]. Other than SEZ and *E. coli*, gram-negative bacteria including *Pseudomonas aeruginosa*, *Klebsiella pneumoniae,* and *Acinetobacter* spp. have also been isolated from jennies with clinical endometritis, although the relative prevalence was relatively low [36]. The treatment of bacterial endometritis commonly used in mares, including uterine lavage, intrauterine antibiotic, ecbolic agents (i.e., oxytocin and prostaglandin), and anti-inflammatory drugs, has been extrapolated in jennies [35].

So far there has not been any documented case of fungal endometritis in jennies. One study recognized the very low frequency of fungal isolation on the external genitalia of jacks, and the fungal species were considered to be airborne contaminants [41]. In mares, fungal endometritis has been related to poor perineal conformation, compromised uterine defenses, necrotic foci, and prolonged antibiotic therapy [42]. It is possible that such correlation applies to jennies as well, but the exact risk factors for fungal endometritis in jennies remain to be determined.

#### 3.1.3. Contagious Equine Metritis and *Taylorella asinigenitalis* Infection

Contagious Equine Metritis (CEM) is a venereal disease in horses caused by gram-negative bacteria *Taylorella equigenitalis* [43]. The World Organization for Animal Health (OIE) classifies it as a reportable disease due to its detrimental effects on equine reproductive health [44]. The typical manifestation in mares is the copious vaginal discharge with temporal infertility, while the stallions usually remain asymptomatic [43]. Experimental infection of these bacteria in the jennies elicited mild, transient clinical signs, although natural infection has not been reported [45]. In recent years, a second *Taylorella* species with great genotypic similarity with *T. equigenitalis* was isolated from the genitals of male donkeys, which was later named as *T. asinigenitalis* [46]. It has been later reported in the U.S. and many European countries [47,48]. *T. asinigenitalis* used to be considered as non-pathogenic, but recently a new strain of *T. asinigenitalis* isolated from a wild jack was reported to cause severe, purulent endometritis in mares [49]. On the other hand, *T. asinigenitalis* does not apparently cause natural diseases in jennies or jacks [50]. A recent study of the donkey population in Spain has revealed a 20.75% positive rate of *T. asinigenitalis* in different Spanish donkey breeds, although no clinical signs were recorded [51].

#### 3.1.4. Endometrosis

The term endometrosis is used to describe the degenerative changes of endometrium typically seen with chronic inflammation in mares [52]. The Kenney and Doig system has been used extensively in the evaluation of endometrial histopathology in mares [53], and it could be applied to jennies due to a lack of a specific grading scheme for the donkey species [54]. The Kenney and Doig system sets four categories (I, IIA, IIB, III) of endometrium based on the level of PMN infiltration, glandular nesting, fibrosis, and stratum compactum integrity [53]. It might require adjustment when applied to the endometrium of jennies which typically contains more neutrophils and eosinophils, potentially leading to a higher-grade classification [6]. Endometrosis is manifested in grade IIA, IIB, and III as fibrotic changes (stromal and/or periglandular) and glandular nesting [55]. In a survey of jennies with endometritis in eastern China, 4.2% (2/48) was classified as grade I, 54.2% (24/48) as grade IIA, 31.2% (15/48) as grade IIB, and 14.6% (7/48) as grade III, suggesting similar pathological changes can occur in the uterus of donkeys [56]. Another study in Portugal found that among the 14 barren jennies, 0 (0%), 6 (42.9%), 5 (36.7%), and 3 (21.4%) jennies were classified as grade I, IIA, IIB, and III [54]. The pathogenesis of endometrosis is related to the abnormal accumulation of collagen under the effects of aging, immunomodulators, and tissue remodeling factors [15]. Specific collagen types (collagen type 1 and 3, COL1/COL3) and cytokines (mainly pro-fibrotic IL-33) have been proved to be associated with fibrosis development in mares and jennies [6,54]. In one study of Catalonian jennies, a correlation was identified between the neutrophil count and COL1 and COL3 expression, suggesting the role of neutrophils in fibrogenesis in the jenny endometrium [57]. The persistence of neutrophils and NETs may cause damage to the endometrium and COL deposition, which leads to fibrosis establishment [15,58]. Future work is needed to shed light on the pathogenesis and fertility prognosis of endometrosis in jennies.

### 3.2. Ovarian Disorders

#### 3.2.1. Anovulatory Follicles

**Figure 3:**
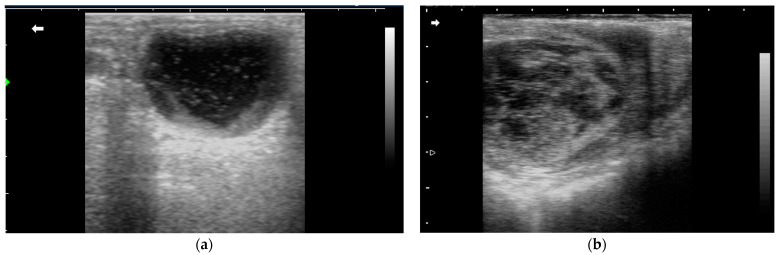
Ultrasound images of hemorrhagic anovulatory follicles (HAFs) in jennies. (**a**) HAFs with small ecogenic dots; (**b**) HAFs with echogenic fibrous bands.

The follicular development pattern in donkeys is similar to mares. Jennies may have single or multiple follicular waves during an estrous cycle, and follicular deviation occurs when the dominant follicle reaches 19–20 mm in diameter at 14–17 days post-ovulation [6,8,59]. The growth rate and the ovulatory diameter of the dominant follicle typically range from 2–4 mm/d and 35–45 mm depending on the breed [6,59]. The development of hemorrhagic anovulatory follicles (HAFs) (Figure 3) has been documented in multiple donkey breeds (Table 3). Like in the mares, HAFs in the jennies are characterized by echogenic fibrin strands within the follicular lumen upon a transrectal ultrasound exam [60]. HAFs were reported to occur in 4.6% (49/1083) of total cycles in Dezhou Black donkeys, a native breed in eastern China, with no significant seasonal variation [59]. In another study about the ovarian dynamics of Caribbean donkeys, HAFs were observed in 1 out of 31 cycles, with ovulation happening in 19 days and normal cyclicity afterwards [61,62]. Another report about HAFs in Catalonian jennies also recorded the spontaneous resolution of the condition with no significant impact on fertility [63]. In mares, metabolic diseases including equine metabolic syndrome (EMS) and pars pituitary intermedia dysfunction (PPID) have been associated with subfertility and anovulatory follicle development [64,65]. Although these conditions have been reported in donkeys, no correlation has been made between the diseases with HAFs formation [66]. Further research is needed to identify the exact mechanism as well as risk factors for anovulatory follicles in jennies.

#### 3.2.2. Other Ovarian Abnormalities

Information about the structural abnormalities of the jenny ovaries is quite limited. One retrospective study about the post-mortem changes of 1444 aged donkeys documented some benign conditions of the ovary including hemangioma, thecoma, and granulosa cell tumor [67]. Another study about slaughtered and necropsy jennies in Egypt revealed 34/83 (40.96%) jennies with ovarian pathological structures including follicular cysts (14/34), paraovarian cysts (1/34), cavernous hemangioma (3/34), granulosa cell tumors (4/34 unilateral, 1/34 bilateral), ovarian endometriosis (4/34), ovarian hematoma (1/34), haemosidrosis (8/34), and oophoritis (5/34) [68]. Ovarian cysts have been reported separately to coincide with polycystic kidney disease in two donkeys in Iran and Qatar, respectively, while the genetic background of the condition remains unclear [69,70]. The influence of abnormal ovarian structures on the fertility of jennies needs to be further investigated.

### 3.3. Abortion

**Figure 4:**
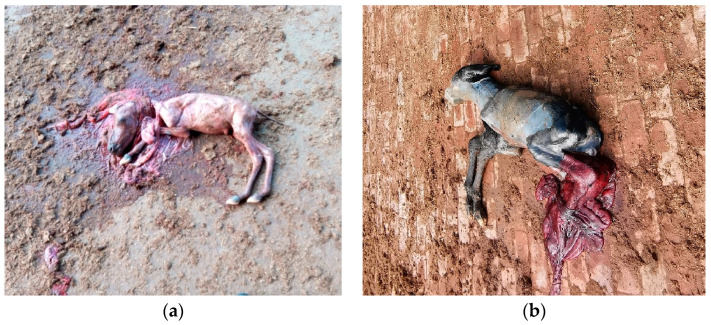
Abortion in jennies. (**a**) Second trimester abortion in a jenny; (**b**) near full-term abortion in a jenny.

While documentation regarding the causes of abortion in jennies (Figure 4) is not as extensive as that for mares, emerging evidence has been adding up towards a more comprehensive understanding of this condition in donkeys. In general, the same diagnostic framework in mares applies to abortion in jennies [71]. Apart from a regular physical exam, rectal palpation, transrectal ultrasound, and vaginal exam of the jenny, the aborted fetus as well as the fetal membranes (if present) should be carefully examined and submitted for laboratory diagnostics including histology, PCR, bacterial culture, and more [72]. A variety of viruses, bacteria, and protozoa have been associated with abortion in jennies (Table 4). Many pathogens can cause abortion in either mares or jennies, but specific agents may exhibit differences in susceptibility between the two species. Infectious causes should be considered in the event of an abortion storm. Like in mares, an exact reason for abortion in jennies may not be identified, especially in underdeveoped areas where diagnostic resources are limited.

#### 3.3.1. Viral Agents of Abortion

Herpesvirus is one of the most common viral causes of abortion reported in jennies. EHV-1 and EHV-4 are the most economically and clinically relevant herpesviruses, which have been associated with fever, respiratory and neurological signs, as well as abortion [83]. EHVs can be transmitted through multiple pathways including direct and indirect contact, and hosts may enter a characteristic latency period after infection [84]. Donkeys can be infected with not only equine herpesviruses including EHV-1 and EHV-4, but also asinine herpesviruses including AHV-1 (EHV-6), AHV-2 (EHV-7), AHV-3 (EHV-8), AHV-4, AHV-5, and AHV-6 [85]. Epidemiological studies of herpesviruses in donkeys have been carried out in various locations throughout the world [86], and it is not uncommon for seropositive animals to remain asymptomatic [75,87,88]. EHV-1 is considered the major abortogenic herpesvirus in horses, and its pathogenicity on pregnancy in donkeys has been confirmed by two separate studies in Egypt and China [73,74]. The abortion events uniformly happened in the last trimester, with the presence of typical EHV-1 pathological changes including vasculitis, pulmonary edema, intranuclear inclusion body, and necrotic changes in multiple organs of the aborted fetus [73]. EHV-4 was found to cause severe upper respiratory tract diseases and an abortion storm in a donkey herd in Romania, where 10 out of 37 symptomatic animals either aborted full-term fetuses or had weak foals that died a few hours after birth [75]. Late-term abortion caused by EHV-7 (AHV-2) and EHV-8 (AHV-3) in jennies has also been documented by sporadic case reports [76,77]. Currently, there is no EHV/AHV vaccine formulated for donkeys, and little is known about the efficacy of commercial EHV vaccines in donkeys [89]. An autogenous inactivated EHV-1 vaccine has been reported to increase antibody titer in donkeys, but the sample size was small (n = 3) and the protective effect was undermined [90].

Other than herpesviruses, *Alphaarterivirus equid* (also known as equine arteritis virus, EAV) is another economically important viral agent that can cause abortion in mares [91]. However, donkeys appear to be more resistant to EAV infection, mostly staying asymptomatic or showing mild clinical signs including transient fever, nasal discharge, and conjunctivitis [86,92,93]. Currently, there is no consensus if the regulatory testing of EAV should be performed before donkey breeding. Future work is needed to elucidate the shedding status of seropositive donkey jacks and the role of venereal transmission in the perpetuation of the virus in donkey populations. Equine Infectious Anemia Virus (EIAV) is another abortogenic virus in mares, but it seems not able to produce diseases in donkeys even though anti-EIA antibodies are developed [94,95]. These may explain the absence of reports of EAV or EIAV-related abortions in donkeys.

#### 3.3.2. Bacterial Agents of Abortion

*Salmonella enterica* subspecies *enterica* serovar abortus equi (*S. abortus* equi) has been a leading cause in the abortion of jennies in China in the last decade [96]. In one survey, 115 out of 122 (94.3%) submitted aborted fetuses tested positive for *S. abortus* equi. In another report, all 45 (22.5%) aborted fetuses from a total of 200 pregnant jennies tested positive while 80% of the donkey population was serologically positive. Infected jennies may abort at any stage of gestation with or without preceding clinical signs, but most aborted fetuses are aged between 200 and 300 days [35,78,79]. Documented gross lesions of the fetuses include splenic hemorrhage, hepatic and nephrotic necrosis, pulmonary fibrosis, and hemorrhagic enteritis, although many aborted fetuses may have no overt lesions grossly [78,79]. *S. abortus* equi has also been reported to cause abortion in mares in many other countries in Europe, South America, and Asia [97,98,99]. The prevention of pregnancy loss from S. abortus equi infection mostly relies on biosecurity surveillance and vaccination due to a lack of effective treatment [78].

Brucellosis is another bacterial disease with the potential to affect the reproductive performance of many domestic animals. Although the presence of Brucella antibodies has been detected in donkey populations in many countries [100,101], so far only one abortion event in a jenny has been documented by a case report in 1968 [80]. In fact, Brucella abortus is a rare cause of abortion in horses as the lesions usually remain localized [102]. *Leptospira* spp. are zoonotic spirochete bacteria that can cause abortion in horses worldwide. Although the seroprevalence can be high in many areas of the world [103,104], no abortion cases related to *Leptospira* spp. have been reported in donkeys. *Enterobacter agglomerans* and β-hemolytic *Streptococcus* have also been sporadically reported to cause abortion in jennies, which may be related to ascending placentitis [35,81,105].

#### 3.3.3. Protozoal Agents of Abortion

Protozoa that can cause abortion in mares include *Neospora* spp., *Theileria equi*, *Babesia caballi*, and *Encephalitozoon cuniculi* [106,107,108]. However, *Neospora caninum* is the only protozoan that has been reported to cause abortion in jennies [82]. In the particular study in Iran, the overall molecular prevalence in jennies’ blood samples and the aborted fetuses was 34.5% (10/29) and 13.8% (4/29), respectively, while transplacental transmission was detected in 40% of aborting jennies [82]. Since little is known about the effective treatment for *Neospora* infection in donkeys, preventative strategies including higher sanitary standards and protection of the feed/water source from oocyte contamination are generally recommended [109].

#### 3.3.4. Non-Infectious Causes of Abortion

**Figure 5:**
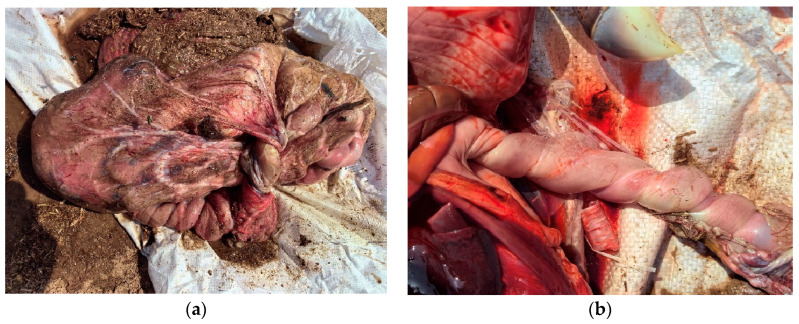
Abortion caused by umbilical cord torsion in a jenny. (**a**) The aborted fetus with covering chorioallantois; (**b**) close-up view of the twisted umbilical cord.

Non-infectious causes of equine abortion include abnormalities of umbilical cord/placenta, poor nutrition or stress response of the dam, twin pregnancy, plant/insect intoxication, and fetal malformations [110]. There is a great likelihood that jennies may suffer from similar conditions to mares, but unfortunately the related literature has been scarce. Umbilical cord torsion (Figure 5) has been reported as the most common non-infectious cause of abortion in mares in a retrospective study [111]. One case of umbilical cord torsion was reported in a 9-year-old donkey where the 8-month-old fetus was found dead in utero [112].

#### 3.3.5. Placentitis

Combined Thickness of Uterus and Placenta (CTUP) from transrectal or transabdominal ultrasonography is a useful screening method for placentitis diagnosis in mares, and its application in monitoring donkey pregnancy has been investigated [9,113]. Similar in the mares, an increase in CTUP with gestation age has been observed in jennies, although variations exist among different breeds [9,113,114]. Limited information is available about the relationship between pregnancy loss and changes in CTUP. While one study about Martina Franca donkeys did not see any changes in CTUP before abortion [114], the other two studies about the Dezhou donkey and Amiata donkeys observed significantly higher values of CTUP with impending abortion [9,113]. Further research is necessary to provide a comprehensive diagnostic plan for placentitis in jennies.

### 3.4. Twin Pregnancy

Twin pregnancy is one of the major causes for pregnancy loss in horses, and accounts for 3% of reported abortions even with intense breeding management [115]. Twinning in equids is usually a result of the fertilization of double ovulation instead of the division of the embryo, and therefore the resultant twins are typically non-identical [116]. Multiple ovulation appears to be common in donkeys, and the double ovulation rate has been reported to be 16–34% and 19.5–26.4% in Dezhou Black donkeys in two different studies [59,117]. The double ovulation rate in Catalonian donkeys was reported to be 42.45%, and vairous levels of tendency for multiple ovulation were observed in different jennies [63]. In Mammoth donkeys, the double ovulation rate can be as high as 70% [118]. The collective information suggests breed and genetic influence on the double ovulation rate in jennies, which is consistent with observation in mares [116]. However, a more in-depth study is needed to reveal the prevalence of twin pregnancy and the occurrence of spontansous reduction in jennies.

Unattended twin pregnancy has been associated with late-term abortion, stillborn, and weak neonates [119]. In one study about Asinina de Miranda donkeys, full-term twin foaling acoounted for 2.85% of total foaling events with a neonatal mortality rate at 40% [120]. In another study about Amiata donkeys, only two pairs of twins were delivered out of 42 pregnancies, and all the foals survived although two (50%) of them developed signs related to mild hypoxic-ischemic encephalopathy [121]. Viable twin foals have also been documented in Martina Franca and some Spanish donkey breeds [122,123].

**Figure 6:**
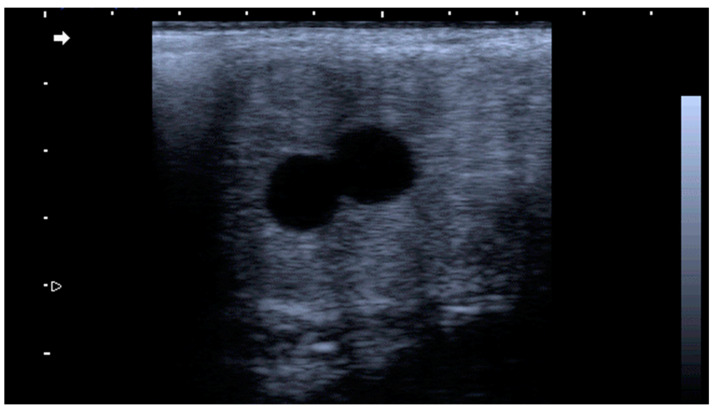
Twin pregnancy (two adjacent embryonic vesicles) detected at 14 days in a jenny.

The routine diagnosis of twin pregnancy is made at 14–16 days of gestation when the embryonic vesicle become readily visble on a transrectal ulrtrasound examination of the uterus [116] (Figure 6). The manual reduction of one vesicle through a transrectal ultrasound has been reported to be a feasible measure to resolve twin pregnancy in jennies [124].

### 3.5. Other Reproductive Disorders of the Jenny

Reports of problems during foaling and post-partum period in jennies are scant. Miscellaneous case reports of other reproductive disorders are summarized in Table 5.

## 4. Reproductive Disorders of the Jack

The genital tract of donkey jacks is generally larger than stallions [6]. Very little information is available about reproductive disorders of the jack. Unilateral cryptorchidism has been documented in three healthy adult donkey jacks, leading to a slight reduction in the functions of accessory sex glands [128]. Diphallia (double penises) and double scrota, a rare congenital disorder of the external genitalia, has also been described [129]. One case of ampullary spermastasis has been reported in a 4-year-old Pêga breed donkey jack which was successfully treated by ampullae massage [130]. Epididymal granuloma has been described in feral donkeys which resulted in an increased level of antisperm antibodies (ASAs), although its impact on fertility remained unknown [131]. In one study about the post-mortem findings of 1444 aged donkeys, no lesion was identified on the male reproductive tract despite careful examination [67]. In another survey about neoplastic conditions in donkeys in North America, sarcoids have been documented at the preputial and scrotal regions in multiple donkey jacks (including geldings) [132]. Although very common in horses, no report of genital squamous cell carcinoma has been documented in donkey jacks, or any organ system of the donkey species [67,132]. Further studies are required for an integral understanding of reproductive disorders in donkey jacks.

## 5. Limitations and Future Directions

One of the primary limitations encountered in this review is the scarcity of comprehensive data on donkey reproductive disorders. Compared to more commonly studied species like horses and cattle, there is a lack of extensive research, which hinders the ability to dive deep into the disease process and draw robust conclusions. In addition, the incidence and management of reproductive disorders in donkeys can vary significantly across different regions. This review might not have captured all regional variations due to the limited availability of region-specific studies. Future research should aim to standardize the diagnostic criteria and methodologies used in the studies of donkey reproductive disorders, which could facilitate more accurate comparisons between studies and improve the reliability of conclusions. More large-scale, multi-regional epidemiological studies are required to better understand the prevalence and risk factors associated with certain reproductive disorders in donkeys. An investigation of the molecular mechanism of the diseases as well as the development or validation of therapy modalities would provide a more solid basis for donkey-specific treatment of reproductive disorders. Collaborative efforts are needed from the researchers, veterinarians, animal owners, and local authorities to improve understanding of reproductive disorders in donkeys towards the better health and welfare of the species.

## 6. Conclusions

In conclusion, this review has underscored the complex landscape of reproductive disorders affecting donkeys, highlighting their unique susceptibility to certain diseases compared with mares and the myriad factors contributing to these conditions. From infertility to infectious diseases and management-related issues, each disorder presents distinct challenges requiring tailored diagnostic and therapeutic approaches. Research gaps persist, particularly in understanding the interactions between genetic predisposition, environmental factors, and reproductive health in donkeys. Moving forward, concerted efforts in clinical practice, research, and education are essential to improve the welfare and reproductive outcomes of this species, ensuring their sustainable contribution to agriculture, conservation, and companion roles worldwide.

## Figures and Tables

**Table 1:** Summary of studies about response to select treatments in jennies with endometritis.

Treatment	Sample Size	Response	Reference
Ketoprofen	6	Decreased COX-2 expression but did not decrease endometrial PMN	[25]
Dexamethasone	6	Did not decrease endometrial PMN but decreased eosinophils 24 h after insemination	[26]
Intrauterine platelet-rich plasma (PRP)	10	Decreased PMN, decreased bacterial isolates, and decreased oxidative stress biomarkers	[28]
	2	Infertile jennies were inseminated and became pregnant	[29]

**Table 2:** Common bacteria isolated in jennies with clinical endometritis reported in the literature.

Bacteria	Percent Positive	Bacterial Identification Method	Sample Size	Country	Reference
*Escherichia coli*	100%	Blood and MacConkey agar, MALDI-TOF MS	10	China	[40]
	50%	Bacterial culture (unspecified)	10	Egypt	[28]
	77%	Blood and MacConkey agar, PCR, MALDI-TOF MS	30	China	[36]
	2%	EMB agar, PCR	123	China	[35]
*Streptococcus equi* subsp. *zooepidemicus*	80%	Bacterial culture (unspecified)	10	Egypt	[28]
	100%	Blood agar, PCR, MALDI-TOF MS	17	China	[34]
	10%	Blood and MacConkey agar, PCR, MALDI-TOF MS	30	China	[36]
	78%	Blood agar, PCR, MALDI-TOF MS	123	China	[35]
*Streptococcus* spp.	70%	Bacterial culture (unspecified)	10	Egypt	[28]
*Acinetobacter* spp.	40%	Blood and MacConkey agar, PCR, MALDI-TOF MS	30	China	[36]
*Pseudomonas aeruginosa*	23%	Blood and MacConkey agar, PCR, MALDI-TOF MS	30	China	[36]
*Klebsiella pneumoniae*	7%	Blood and MacConkey agar, PCR, MALDI-TOF MS	30	China	[36]

EMB agar = Eosin-Methylene Blue agar; MALDI-TOF MS = matrix-assisted laser desorption ionization-time of flight mass spectrometry.

**Table 3:** Reports of hemorrhagic anovulatory follicles (HAFs) in different donkey breeds.

Donkey Breed	Location	Cycle Number	HAF Number (%)	Outcome	Reference
Dezhou Black donkey	China	1083	49 (4.6%)	Unspecified	[59]
Caribbean donkey	West Indies	31	1 (3.2%)	Ovulation in 19 days (contralateral ovary)	[61,62]
Catalonian donkey	Spain	14	2 (14.3%) in the same jenny	Ovulation in 23 and 46 days, respectively	[63]

**Table 4:** Summary of reported infectious agents causing abortion in jennies.

Pathogen Type	Pathogen	Sample Size ^1^	Breed	Location	Clinical Signs	Reference
Virus	EHV-1	1	Unspecified	Egypt	3rd trimester abortion	[73]
		3	Unspecified	Xinjiang, China	Abortion storm (3rd trimester) affecting 40% (80/200) of pregnant donkeys	[74]
	EHV-4	10	Unspecified	Romania	Abortion storm (stillborns/foals dead soon after birth) in 27% (10/37) animals showing respiratory signs	[75]
	EHV-7 (AHV-2)	1	Mediterranean miniature donkey	Washington, US	3rd trimester abortion	[76]
	EHV-8 (AHV-3)	1	Unspecified	Shandong, China	3rd trimester abortion	[77]
Bacteria	*Salmonella abortus*	9	Dezhou Black, Qingyang, and Xinjiang donkey	Eastern China	Abortion storm affecting 12% (61/500) of pregnant donkeys	[78]
		45	Unspecified	Shandong, China	Abortion storm affecting 22.5% (45/200) of pregnant donkeys	[79]
		115	Unspecified	China	Abortion storm affecting 24.4% (427/1753) of pregnant donkeys on multiple farms	[35]
	*Brucella abortus*	1	Unspecified	UK	3rd trimester abortion	[80]
	*Enterobacter agglomerans*	1	Unspecified	India	3rd trimester abortion	[81]
	*Streptococcus* spp.	1	Unspecified	China	Abortion storm affecting 20.8% (5/24) of pregnant donkeys	[35]
Protozoa	*Neospora caninum*	4 (fetus) 10 (jenny)	Iranian donkey	West and North Iran	19/29 abortions happened in 2nd–3rd trimester	[82]

^1^ The number of animals that tested positive for specific pathogens.

**Table 5:** Summary of case reports depicting miscellaneous reproductive disorders of the jenny.

Condition	Signalment	Clinical Abnormalities	Treatment and Outcome	Reference
Dystocia	Full-term pregnant jenny	One flexed forelimb and lateral deviation of head	C-section with dead fetus. Jenny recovered with no complications.	[125]
	5-year-old full-term primiparous jenny	Carpal flexion of both forelimbs and lateral deviation of head	Assisted vaginal delivery with dead fetus	[126]
Fetal maceration	10-year-old jenny	Posterior presentation and posterior limbs flexed to hip	C-section with dead fetus. Jenny returned to estrus in 40 days.	[127]
Retained fetal membranes	6-year-old jenny with normal foaling	Placenta retained for >12 h	Gentle manual traction and successful removal of placenta	[112]

## Data Availability

No new data were created in this study.
